# Development and Implementation of a Decision Support System to Improve Control of Hypertension and Diabetes in a Resource-Constrained Area in Brazil: Mixed Methods Study

**DOI:** 10.2196/18872

**Published:** 2021-01-11

**Authors:** Milena Soriano Marcolino, João Antonio Queiroz Oliveira, Christiane Corrêa Rodrigues Cimini, Junia Xavier Maia, Vânia Soares Oliveira Almeida Pinto, Thábata Queiroz Vivas Sá, Kaique Amancio, Lissandra Coelho, Leonardo Bonisson Ribeiro, Clareci Silva Cardoso, Antonio Luiz Ribeiro

**Affiliations:** 1 Medical School Universidade Federal de Minas Gerais Belo Horizonte Brazil; 2 Telehealth Center, University Hospital Universidade Federal de Minas Gerais Belo Horizonte Brazil; 3 Medical School Universidade Federal dos Vales do Jequitinhonha e Mucuri Teófilo Otoni Brazil; 4 Medical School Universidade Federal de São João del-Rei Divinópolis Brazil

**Keywords:** clinical decision support systems, primary health care, hypertension, diabetes mellitus, evidence-based practice, telemedicine, patient care management

## Abstract

**Background:**

The low levels of control of hypertension and diabetes mellitus are a challenge that requires innovative strategies to surpass barriers of low sources, distance, and quality of health care.

**Objective:**

The aim of this study is to develop a clinical decision support system (CDSS) for diabetes and hypertension management in primary care, to implement it in a resource-constrained region, and to evaluate its usability and health care practitioner satisfaction.

**Methods:**

This mixed methods study is a substudy of HealthRise Brazil Project, a multinational study designed to implement pilot programs to improve screening, diagnosis, management, and control of hypertension and diabetes among underserved communities. Following the identification of gaps in usual care, a team of clinicians established the software functional requirements. Recommendations from evidence-based guidelines were reviewed and organized into a decision algorithm, which bases the CDSS reminders and suggestions. Following pretesting and expert panel assessment, pilot testing was conducted in a quasi-experimental study, which included 34 primary care units of 10 municipalities in a resource-constrained area in Brazil. A Likert-scale questionnaire evaluating perceived feasibility, usability, and utility of the application and professionals’ satisfaction was applied after 6 months. In the end-line assessment, 2 focus groups with primary care physicians and nurses were performed.

**Results:**

A total of 159 reminders and suggestions were created and implemented for the CDSS. At the 6-month assessment, there were 1939 patients registered in the application database and 2160 consultations were performed by primary care teams. Of the 96 health care professionals who were invited for the usability assessment, 26% (25/96) were physicians, 46% (44/96) were nurses, and 28% (27/96) were other health professionals. The questionnaire included 24 items on impressions of feasibility, usability, utility, and satisfaction, and presented global Cronbach α of .93. As for feasibility, all professionals agreed (median scores of 4 or 5) that the application could be used in primary care settings and it could be easily incorporated in work routines, but physicians claimed that the application might have caused significant delays in daily routines. As for usability, overall evaluation was good and it was claimed that the application was easy to understand and use. All professionals agreed that the application was useful (score 4 or 5) to promote prevention, assist treatment, and might improve patient care, and they were overall satisfied with the application (median scores between 4 and 5). In the end-line assessment, there were 4211 patients (94.82% [3993/4211] with hypertension and 24.41% [1028/4211] with diabetes) registered in the application’s database and 7960 consultations were performed by primary health care teams. The 17 participants of the focus groups were consistent to affirm they were very satisfied with the CDSS.

**Conclusions:**

The CDSS was applicable in the context of primary health care settings in low-income regions, with good user satisfaction and potential to improve adherence to evidence-based practices.

## Introduction

Hypertension and diabetes are leading modifiable risk factors for cardiovascular disease worldwide, major contributors to premature disability, and associated with substantial premature death and morbidity [1]. Despite all the advances in the therapy for these diseases, and the fact that many effective treatments are available, there is a great deal of room for improvement. There is a large gap between detection and control of both diseases, and the majority of patients do not reach therapeutic goals [2-4]. The situation is worse in low- and middle-income countries and even worse in rural communities, where awareness, treatment, and control of hypertension are lower than in urban areas [2,4,5].

To face this challenge, innovative strategies are required. Clinical decision support systems (CDSSs), which are capable of generating suggestions or information that is specific to individual patients, based on the unique individualized patient information, can make a significant contribution to the effective dissemination of evidence-based practice. They may increase practitioners’ adherence to guidelines, involving clinicians in the translation of research into practice [6]. Therefore, they may increase the proportion of patients who reach blood pressure and glucose goals, with a potential impact in reducing cardiovascular risk.

However, the evidence of impact of CDSSs on key diabetes care outcomes, such as the control of glucose, blood pressure, tobacco use, or appropriate aspirin use has been marginal or inconsistent. One important barrier in this context is CDSS usability [7]. Many CDSSs were not used regularly or on a sustained basis by primary care physicians [8]. This is true not only for diabetes, but also for CDSS in general. It has been reported that their adoption has been somewhat limited outside of a relatively small number of academic medical centers and integrated health care delivery networks [9,10]. Therefore, in order to translate evidence into practice and develop CDSSs, which can be useful and used in clinical practice, studies that assess usability prior to a large-scale implementation are of utmost importance [11].

Thus, our aim was to develop a CDSS for diabetes and hypertension management in primary care, to implement it in a low-income and mostly rural region, and to evaluate its usability and health care practitioner satisfaction.

## Methods

### Study Overview

This is a substudy of the HealthRise Brazil Project, a quasi-experimental study, which is part of a multinational study designed to implement and evaluate pilot programs aimed at improving screening, diagnosis, management, and control of hypertension and diabetes among underserved communities [12]. Interventions were designed and implemented in 9 communities in Brazil, India, South Africa, and the United States of America. Each program was designed and implemented by a local grantee and included interventions that were tailored to local needs and contexts [12]. HealthRise Brazil Project was conducted in 2 centers, Vitória da Conquista, in the state of Bahia, and in the region of Teófilo Otoni (Vale do Mucuri), the state of Minas Gerais. In this region, the project was implemented in 10 municipalities: Frei Gaspar, Ouro Verde de Minas, Crisólita, Catuji, Setubinha, Itaipé, Novo Oriente de Minas, Ladainha, Teófilo Otoni, and Ataleia.

**Figure 1:**
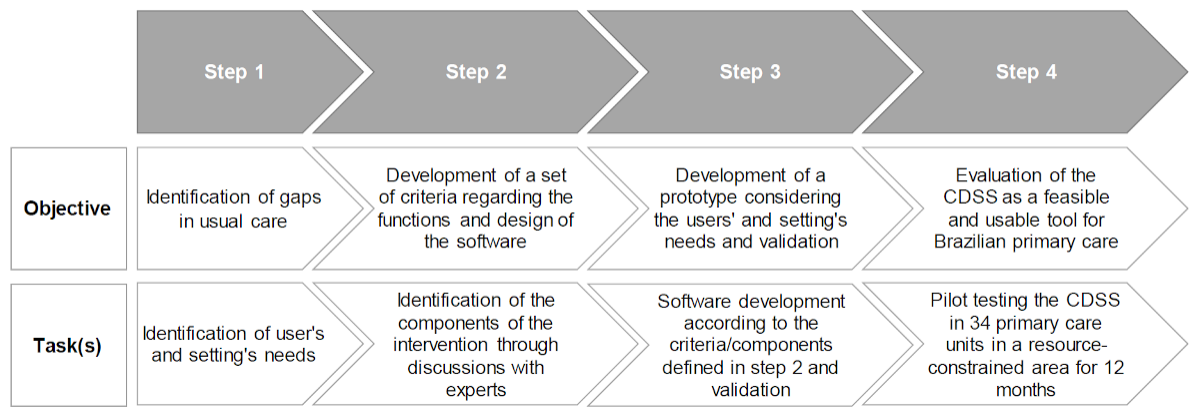
Flowchart of the study methodology. CDSS: clinical decision support system.

This study was conducted by the HealthRise Brazil Teófilo Otoni team in 4 steps, according to the Medical Research Council framework (Figure 1) [13]: (1) identification of gaps in usual care; (2) identification of the components of the intervention through discussions with experts; (3) CDSS development and validation; and (4) pilot testing. Theory of change thinking was used throughout the study: programme design, monitoring, and evaluation [14,15].

### Identification of Gaps in Usual Care

To evaluate which gaps were already known, we assessed: (1) epidemiological studies and systematic reviews on hypertension and diabetes management; (2) secondary data, including census, *Sistema de Informação Ambulatorial* (Outpatient Information System), *Sistema de Informação da Atenção Básica* (Primary Care Information System), *Cadastro Nacional de Estabelecimentos de Saúde* (National Registry of Health Establishments), *Programa Nacional de Melhoria de Acesso e da Qualidade da Atenção Básica* (National Program for Improving Access and Quality of Primary Care), social dimensions of the research “Inequalities,” and *Pesquisa Nacional de Saúde* (National Health Survey). We also assessed information from workshops and focus groups performed by the sponsor, with the participation of municipal and state government health managers, physicians, pharmacists, nurses, nutritionists, physical educators, university professors, specialists, representatives of patients and local communities, community health workers (CHWs), who discussed barriers and access opportunities, as well as priority areas (diagnosis, treatment, and disease management). This information was available the moment we applied for the research funding [12], and it was the starting point to generate ideas for the intervention design [16].

### Identifying the Components of the Intervention Through Discussion With Experts and Stakeholders

In order to identify the components of the intervention, information was derived from the previous step, as well as previous assessments from our group [17,18]; discussions with primary care physicians, cardiologists, endocrinologists, nurses, nutritionists, pharmacists, and physical educators. Besides, meetings and internal workshops were conducted to discuss the topic with primary care practitioners, primary care unit coordinators, local health authorities, and stakeholders. The key stakeholders were identified and involved in the project from the beginning of design of the intervention [15].

A primary care physician, an internal medicine specialist, a cardiologist, and an endocrinologist discussed the gaps with experts in information technology and stakeholders to identify solutions and components of the intervention, to map anticipated change processes, long-term changes that needed to happen in the target group’s lives, the barriers to those changes, and to explore assumptions and hypotheses [16].

At that time, all primary care units used a paper-based system to manage patient records; there were different levels of internet connectivity in the primary care centers and health care workers had low technical literacy. All of these factors were taken into account.

### Clinical Decision Support System Development and Validation

#### Software Requirements

A team of clinicians established the functional software requirements necessary to record, track, and support decision making for patients with hypertension, diabetes, or both, according to the information obtained from the previous step and also from national and international guidelines [19-29] to define the technical specifications for digital systems. A list of indicators was defined for data monitoring.

#### Development of Content and Functionalities

The technologies used for software development were chosen according to the available technological infrastructure in each municipality. In this context, the following aspects were taken into account: (1) existence of internet connection; (2) type of internet connection; (3) quality of internet connection; (4) existence of mobile and landline telephony; (5) equipment available in health units. Given the different realities and often precarious technology and information, the challenges faced were huge.

For the execution of the project, there was an agreement between the mayor of each municipality, the coordinator of each health unit, and the project management team to guarantee a minimum internet connection and the existence and maintenance of a local server network that would guarantee the registration and access to data while assisting patients. Despite this agreement, and because certain locations had few resources and limited or unstable internet connections, the server system was developed to work locally without the need for an internet connection. The internet was required only to synchronize local servers to the central database, whenever there was a connection, for scientific and monitoring purposes. Therefore, small drops in connection would not lead to any data loss or force an abandonment of using the system. With regard to problems in connectivity and transmission, in cases of momentary loss of synchronization of local data with the centralized data pool, data were synchronized whenever the connection was re-established. In remote areas, we used a portable internet router for transmission, and in rare instances of total absence of internet, which impaired the synchronization of the monthly data monitoring, data were copied to a portable storage device. With data from all units transmitted to the central database, these were extracted and exported to an Excel spreadsheet (Microsoft) for analysis by the research team (Multimedia Appendix 1).

The application was developed with an “user-centered design” [15], in Java language 1.8 and JSF 2.2, using NetBeans 8.1 development environment, PostgreSQL database server, and Hibernate framework, to be used in web browsers. The interface was developed to be intuitive and self-explanatory, using Prime Faces, Bootstrap, JavaScript, and jQuery. Security and access control were based on Spring Security, ensuring inviolability of the data. There was an iterative process of development, with close and daily cooperation between clinicians and developers of the application [15].

**Figure 2:**
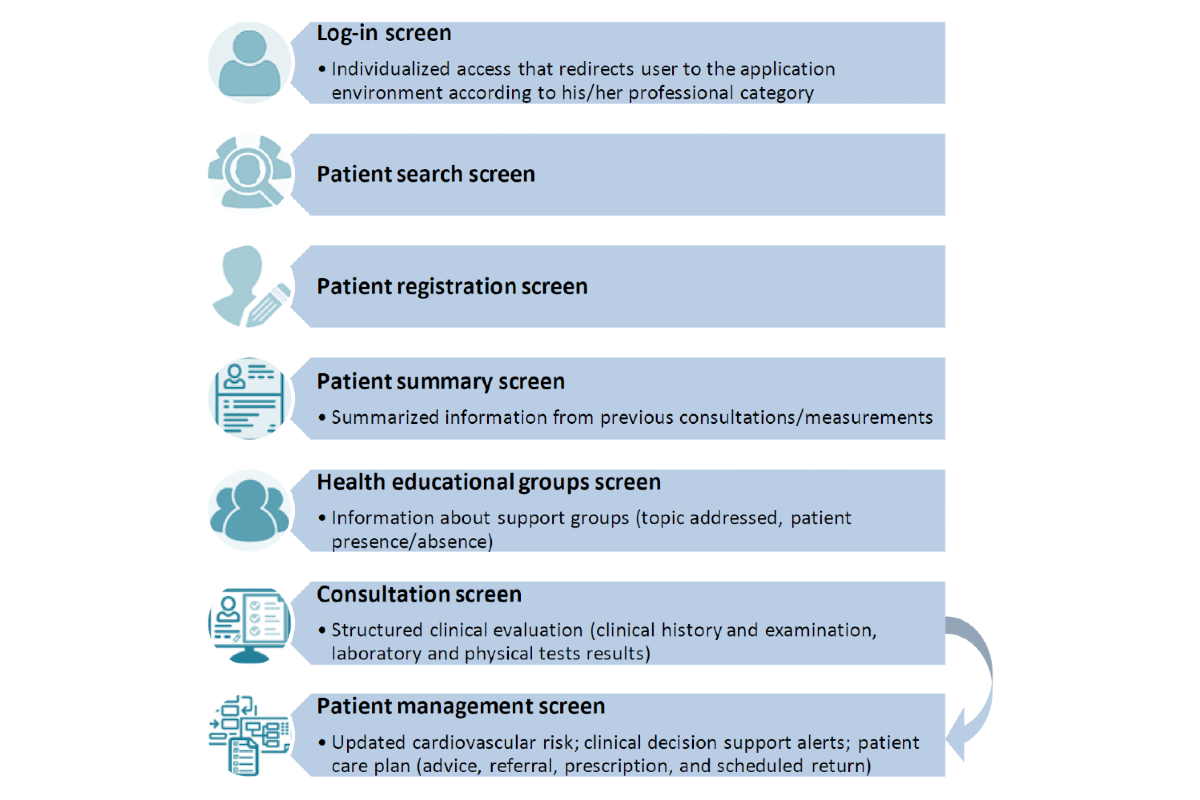
The main functionalities of the application.

The application was designed to be used by health care professionals, and consists of: (1) a log-in screen; (2) a patient search screen; (3) a patient registration screen; (4) structured clinical evaluation, clinical decision support, and patient care plan; (5) a health educational groups screen; (6) patient summary screen; and (7) patient management screen (Figure 2).

The log-in screen allows individualized access to the system through the credentials (user and password) provided to each professional. Each user is registered by a central administrator and each user account is associated with a professional profile that describes the way the application environment looks and operates for that user: physician, nurse, nurse technician, multidisciplinary primary care support team (*Núcleo Ampliado de Saúde da Família e Atenção Básica [*NASF-AB*]*, explained further in this article), and CHW.

After login, the professional has access to the screen to search for registered patients. If the patient was not registered before, he/she can access the registration screen, to register a new patient (demographic data, address, and telephone number). After registration, the other functionalities become available. Each patient has a specific identification code in the database.

The main functionalities of the application consist of the structured clinical evaluation, the clinical decision support, and the patient care plan. Data requested were manually entered, but data previously entered were saved for the next consultation, for example, medications used. In this case, the health professional could edit the information as necessary. Although few fields are mandatory, the application alerts the health care professional to the importance of completing the information appropriately and completely.

**Figure 3:**
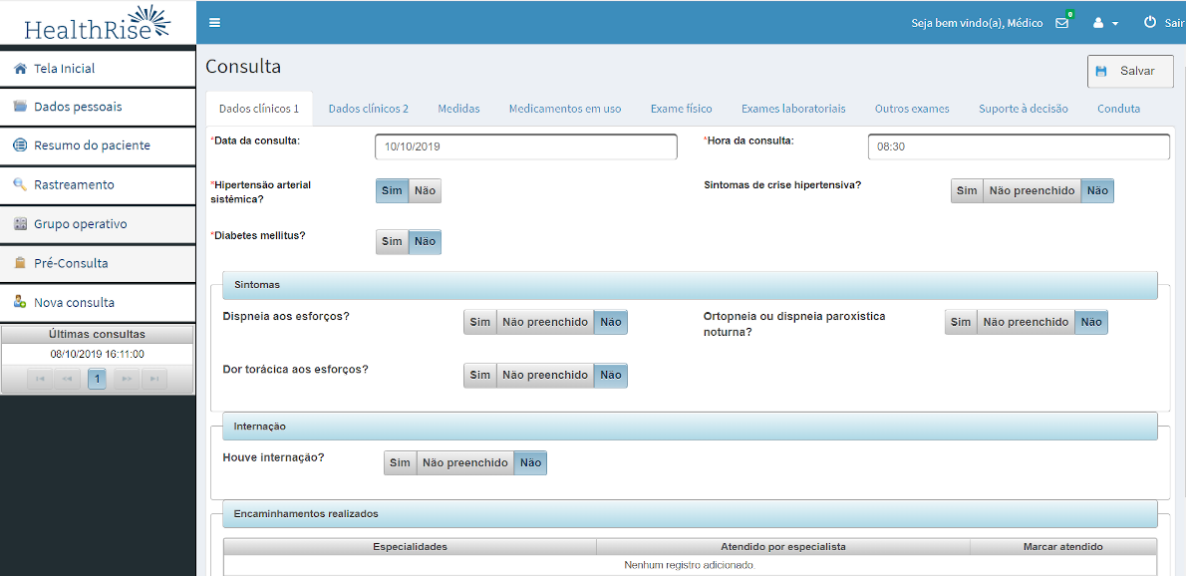
Structured clinical evaluation.

The structured clinical evaluation is shown partially in Figure 3. It includes data on symptoms, medical history, physical examination (including foot examination in patients with diabetes), current medications, and complementary examination results (laboratory and other tests).

To develop the clinical decision support functionality, recommendations of Brazilian and international evidence-based guidelines assessing hypertension, diabetes, cardiovascular risk, and chronic renal disease were reviewed [19-29]. The most relevant clinical recommendations for the management of patients with hypertension, diabetes, or both were derived and organized into a decision algorithm, which bases the CDSS alerts, reminders, and suggestions. In case of conflicting recommendations, the one with the best level of evidence was chosen. When evidence-based guideline recommendations were not available or if they were considered outdated, evidence-based summaries were assessed [30]. Previous experiences of our group were important for the development of this functionality. Suggestions received about usability in previous opportunities were decisive to improve and create new messages and resources [17,18].

**Figure 4:**
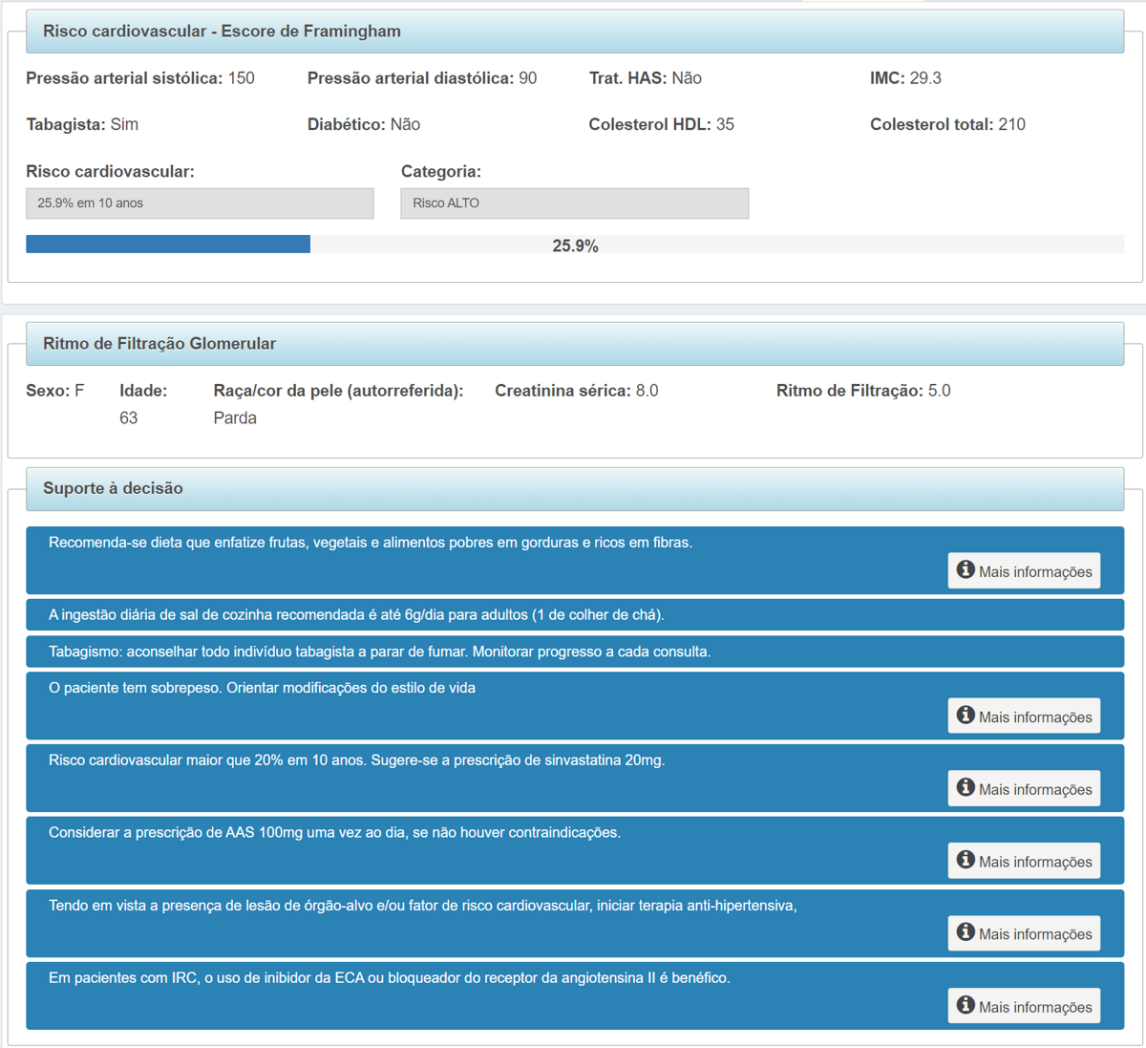
Clinical decision support tab.

The clinical decision support tab is shown in Figure 4. In this tab, health care professionals could also access personalized messages to guide the management of each patient, generated according to the data entered in each consultation.

The following variables were calculated using the data entered: (1) BMI, (2) estimated glomerular filtration rate using the Chronic Kidney Disease Epidemiology Collaboration (CKD-EPI) equation [31], and (3) cardiovascular risk based on the Framingham score [32]. For the alert messages, each one contained a summarized recommendation, tailored according to each patient’s state of health, with auxiliary text containing detailed information and references that support the recommendation.

In the patient care plan, the health care professional can register nonpharmacological interventions, complementary examinations requested, specialist referral and drug prescription (the last 2 functionalities are available only to doctors), and the date for the next consultation. It is also possible to request teleconsultation, defined as a second opinion system that allows an information exchange between distant and local health care professionals, in order to discuss a clinical case when a specialist is not locally available [33]. For this project, it was possible to forward doubts straight to a family physician and an endocrinologist, who were exclusively available for this project, or to other medical and nonmedical specialties, following the workflow of the Telehealth Network of Minas Gerais, a large public Telehealth service which assists 816 municipalities in the state of Minas Gerais [34]. All teleconsultations were asynchronous. The health care professional could choose which specialty he/she was referring the teleconsultation to, and the normal response time did not exceed 48 hours.

**Figure 5:**
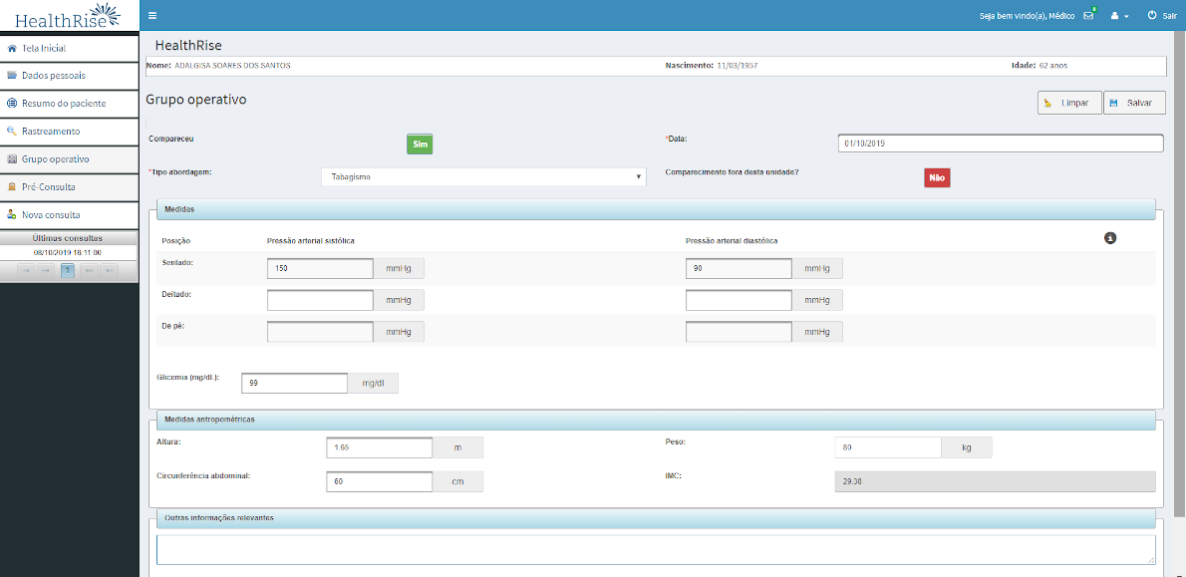
Health educational groups screen.

In the educational health groups (Figure 5) screen, the health care professional can register activities performed in different groups in which that patient belongs to (such as hypertension, diabetes, smoking, nutrition).

Patient data obtained in each group (weight, waist circumference, and blood pressure measurements, as well as capillary blood glucose) can be registered, as well as individual absenteeism. There is also a free-text field for any other information that the health professional believes is necessary.

**Figure 6:**
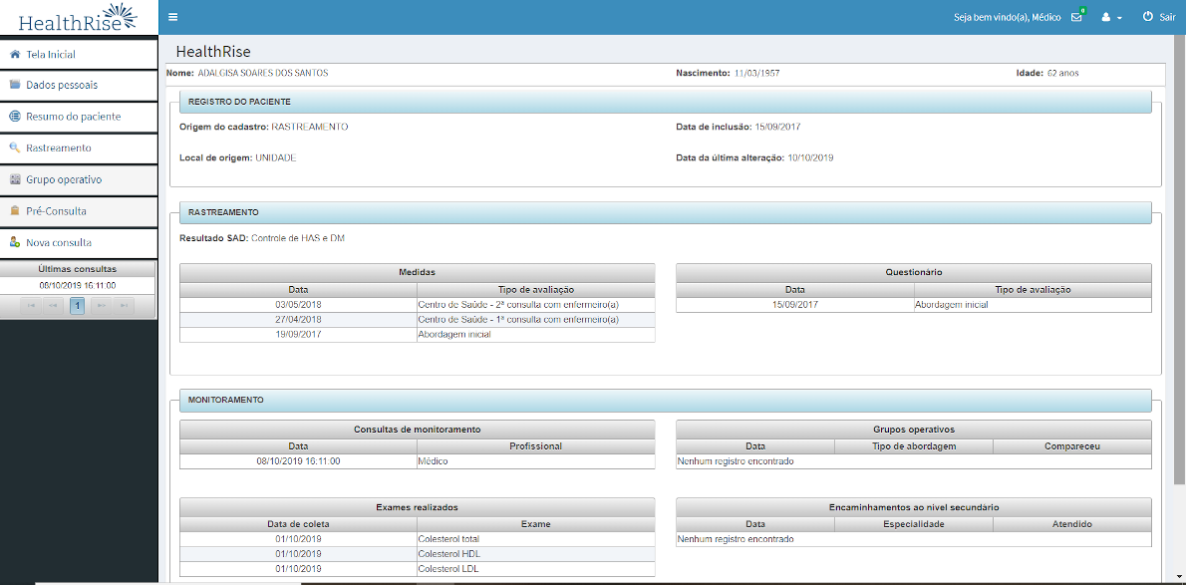
Patient summary screen.

The patient summary screen (Figure 6) shows an overview of the dates of consultations and complementary examinations for each patient.

To conclude the consultation, the health care professional must save the data entered and has the option to print a structured medical record file with all information entered in that consultation, to attach to a physical patient chart. The medical prescription and the referral form can also be printed. Data are recorded in the application and transmitted to a telehealth care center as soon as a stable internet connection was available.

In the patient management screen, it is possible to generate patient lists, according to specific monitoring indicators.

#### Pretesting

The prototype has been tested multiple times with test cases to ensure that the system was operating as intended, free of bugs, and that the recommendation results matched the prespecified decision tree. A manual insertion of data by 2 physicians, 2 pharmacists, 1 nurse, and 3 medical students with test cases was performed to verify the recommendation response suitability.

#### Expert Panel Assessment

After adjustments, the prototype was submitted for expert panel assessment, composed of 2 cardiologists, 1 primary care physician, 1 endocrinologist, 1 nurse, 1 pharmacist, and 1 physiotherapist, known as technical reference, who were independent from the project and its implementers, in order to retain a degree of impartiality [15]. The experts used the application for 2 weeks, and were asked to assess functional suitability, stability, appropriateness of CDSS content, clinical benefit, readability, strengths, inconsistencies, perception of usefulness, whether it was appropriate to the local context, and satisfaction with the application. The prototype was then re-adjusted with the necessary changes, according to feedback from the expert panel.

### CDSS Implementation

The field study was conducted from October 17 to October 18 in 5 primary care centers in Teófilo Otoni, the main city in the northeast region of Minas Gerais, with 141,934 inhabitants [35], and 29 primary centers in 9 small towns (with population less than 20,000 inhabitants), as part of the HealthRise Brazil Project—Vale do Mucuri. The region has a low human development: a mean Human Development Index (HDI) of 0.701 for Teófilo Otoni and 0.540-0.595 for the other municipalities, similar to poor African countries. About one-third of the population lives in rural areas (32.3%) [36]. The HealthRise Brazil Project (2016-2018) consists of 2 parts: patient screening and management (logic model is shown in Multimedia Appendix 2).

The intervention was multifaceted, including extensive training of primary care teams, empowerment of patients through education, improvements in access to examinations, strengthening of health educational groups, and implementation of the computer decision support application.

This study is part of patient management. For this substudy, 34 primary health centers in urban and rural areas were included, with family health teams composed of 1 physician and 1 nurse, 1 nurse assistant, and 4 to 6 full-time CHWs [37]. Family health service (FHS) teams provided comprehensive, universal primary care to defined geographical catchment areas, covering populations of up to 1000 households each (between 3000 and 4000 inhabitants), with no overlap or gap between catchment areas. Each FHS team member had defined roles and responsibilities, and national guidelines helped structure FHS responses to most health problems [37,38]. Pharmacists, physiotherapists, nutritionists, psychologists, physical education specialists, and social workers were part of the multidisciplinary primary care support teams (NASF-AB). Each NASF-AB team assisted 3 or 4 primary care centers [37]. Despite the existence of a university in Teófilo Otoni, clinicians have limited access to specialist referrals and to continuous learning.

Local health authorities and stakeholders were involved in the implementation of the system. The implementation of the application was stepwise, following a schedule of in-person and practical trainings. The participants had the opportunity to handle the devices that would be used. Different training strategies were developed for each professional category. The participants received printed material for further consultation in case of doubt. Furthermore, an online tutorial about the topic was recorded and was available on a platform where they could access online classes about different topics such as hypertension and diabetes management, cardiovascular risk, and operative group planning and organization. Two technology technicians were available online and by telephone to solve doubts and difficulties that arose while using the application. If necessary, they were able to visit each unit.

Fidelity was monitored periodically through checking whether field implementation altered the functionality and stability of the system, changing the intervention from that which was intended, as recommended by the World Health Organization [15]. Malfunctions and other problems with use of the CDSS were quickly fixed.

### Feasibility, Usability, and Satisfaction Assessment

A questionnaire evaluating perceived feasibility, usability, and utility of the application and professionals’ satisfaction, previously developed by our group [18], was applied after 6 months. The first part of the questionnaire included participant characteristics: age, specialty, time since graduation, time working in primary care, previous knowledge in dealing with health technologies, previous access to the internet, and previous access to continued education. The second part included Likert scale questions, varying from 1 (strongly disagree) to 5 (strongly agree), to assess feasibility, usability, and satisfaction. This instrument was evaluated in its reliability capacity applied in this context.

At the end of the HealthRise Project, 2 focus groups with primary care physicians and nurses were performed. The health care professionals talked about the advantages of using the software and what they thought should be improved. The sessions lasted for 1.5 hours and were audio-recorded. Questions started out broadly and became more specific as participants felt more comfortable and opened up.

### Data Analysis

Distributions of continuous variables were examined for normality by using Kolmogorov–Smirnov tests. As the distributions were asymmetrical, these variables were expressed as median and corresponding IQR. Categorical variables were presented as frequencies and proportions. The reliability of the questionnaire to evaluate the perceived feasibility, usability, and utility was evaluated using Cronbach α. Data analyses were performed using the software IBM SPSS Statistics for Windows, version 21.0 (IBM Corp.).

### Ethics

Ethical approval was obtained from the Universidade Federal dos Vales do Jequitinhonha e Mucuri Research Ethics Committee (number 65808517.9.0000.5108). Written and informed consent was obtained from all participants.

## Results

### Phase I: CDSS Development and Validation

An initial bank of 168 reminders and suggestions was created for the clinical decision support functionality. After message content refinement, a final bank of 159 reminders and suggestions was used. Textbox 1 shows examples of the CDSS messages. As there were many messages, we choose to extensively test 26 messages first; we implemented the application with those messages, and continued testing the other messages extensively. We divided the messages in blocks of 10, ranked the groups according to the priority of the recommendations for diabetes and hypertension management, and tested each group before implementation.

**Examples of clinical decision support system reminders and suggestions. ACE: angiotensin-converting enzyme.**

- “Reinforce the importance of adherence to treatment and participation in the educational group. Request a visit from the community health worker”.
- “Cardiovascular risk greater than 20% in 10 years. The prescription of statin is suggested.”
- “Do not prescribe thiazide diuretic for this patient due to reduced renal function.”
- “If you decide to add another anti-hypertensive, choose one of the first-line groups (ACE inhibitor, angiotensin receptor II blocker, thiazide diuretic, long-acting calcium channel blocker), taking the contraindications into account.”
- “Metformin is being used at a dosage above the maximum recommendation. Dose reduction is suggested.”
- “This patient did not have a fundoscopic assessment in the last year”.
- “It is suggested to schedule the next consultation in one month, to reassess blood pressure levels”.

During internal tests, most errors identified were due to misplaced parenthesis and connectors in the commands of the decision algorithm. Posterior manual insertion of data evidenced remaining errors, spelling mistakes, and inconsistencies that were properly revised before the test by the panel of specialists. The expert panel–suggested changes were used to enhance the application.

### Phase II: Feasibility, Usability, and Satisfaction Assessment

#### Quantitative Analysis

At the 6-month assessment, there were 1939 patients registered in the application database and 2160 consultations were performed by primary care teams. Of the 96 health care professionals who were invited for the usability assessment, 26% (25/96) were physicians, 46% (44/96) were nurses, and 28% (27/96) were from other health professionals. The number of participants per town varied from 5 (in Crisólita) to 14 (in Ataleia). Participant characteristics are shown in Table 1. Age varied from 23 to 68 and time since graduation varied from 1 to 29 years. Nurses and other health professionals were predominately women.

**Table 1:** Characteristics of health care professionals who participated in the study (N=96).

Variable		Physicians (n=25)	Nurses (n=44)	Other health professionals (n=27)	Total
Age (years), median (IQR)		35 (30-43)	33 (30-36)	32 (27-36)	33 (30-36)
Female sex, n (%)		11 (44)	33 (75)	22 (81)	66 (69)
Time since graduation (years), median (IQR)		9.0 (7.0-11.0)	9.0 (6.5-11.0)	5.5 (3.0-9.0)	8.0 (4.3-10.8)
Time working in primary care (years), median (IQR)		7.0 (4.0-16.0)	5.5 (2.0-9.4)	3.0 (1.0-4.0)	5.0 (2.0-9.0)
**Self-reported knowledge in information technology, n (%)**					
	Excellent	9 (36)	10 (23)	7 (26)	26 (27)
	Good	9 (36)	24 (55)	15 (56)	48 (50)
	Satisfactory	7 (28)	9 (20)	5 (19)	21 (22)
	Inadequate	0 (0)	1 (2)	0 (0)	1 (1)
Use of any form of technology for work before this research project (yes), n (%)		12 (48)	31 (70)	19 (70)	62 (65)
Computer available in the workplace for routine use before the research project (yes), n (%)		11 (44)	32 (73)	19 (70)	62 (65)
Internet access in the workplace (yes), n (%)		20 (80)	39 (89)	23 (85)	82 (85)
**Internet use frequency (yes), n (%)**					
	Daily	21 (84)	40 (91)	22 (81)	83 (86)
	Almost daily	2 (8)	3 (7)	4 (15)	9 (9)
	Weekly	2 (8)	0 (0)	0 (0)	2 (2)
	Rarely	0 (0)	1 (2)	0 (0)	1 (1)
Participated in an updating activity on management of hypertension, diabetes mellitus, or cardiovascular risk in the last year (yes), n (%)		17 (68)	18 (41)	7 (26)	42 (44)

The results of the questionnaire including 24 items on impressions of feasibility, usability, and utility are presented in Table 2. The reliability analysis of the instrument and 4 dimensions, applied to this population, presented a global Cronbach α of .93 (Table 2 and detailed in Multimedia Appendix 3), showing adequate internal consistency of this instrument for evaluation of the perceived feasibility, usability, and utility of the CDSS.

**Table 2:** Feasibility, usability, and satisfaction assessment scoresa (N=96).b

Item		Physicians (n=25)	Nurses (n=44)	NASF-AB^c^ (n=27)	Total
**Feasibility, median (IQR)**					
	The application can be used in primary care setting	4 (4-5)	5.00 (4-5)	4 (4-5)	4 (4-5)
	It is easy to be incorporated in work routine	4 (2-5)	4 (4-4)	4 (3-4)	4 (3-4)
	It does not cause significant delays in daily routine	2 (2-3)	3 (2-4)	4 (3-4)	3 (2-4)
**Usability, median (IQR)**					
	Overall, evaluation is good	4 (3.25-4.75)	4 (4-5)	4 (4-5)	4 (4-5)
	The screens are easy to understand	4 (4-4)	4 (4-5)	4 (4-4.5)	4 (4-5)
	I was able to find the information I was looking for	4 (4-4)	4 (4-4)	4 (4-5)	4 (4-4)
	The definitions of comorbidities are clear	4 (4-4)	4 (4-4.75)	4 (4-5)	4 (4-4)
	The fields are easy to fill	4 (4-4)	4 (4-5)	4 (4-5)	4 (4-5)
	It has proper interface	4 (4-4)	4 (4-5)	4 (4-5)	4 (4-5)
	It is stable during the use	4 (2-4)	3 (2-4)	3 (2-4)	3 (2-4)
**Utility, median (IQR)**					
	The application might improve patient care	4 (4-5)	5 (4-5)	5 (4-5)	4 (4-5)
	The recommendations brought new information on hypertension, diabetes mellitus, and cardiovascular risk	4 (4-4)	4 (4-5)	4 (4-5)	4 (4-5)
	It was useful to calculate the cardiovascular risk	4 (4-4.75)	4 (4-5)	4 (4-5)	4 (4-5)
	It was useful to promote cardiovascular disease prevention	4 (4-5)	4 (4-5)	4 (4-5)	4 (4-5)
	It assisted me to treat my patients	4 (4-4)	4 (4-5)	4 (4-4)	4 (4-5)
	It assisted me to choose complementary examinations	4 (3-4)	4 (4-5)	4 (3.75-4.25)	4 (4-5)
	It was helpful to decrease referral to specialists	4 (2-4)	4 (3-4)	4 (4-4)	4 (3-4)
	It was useful for different professional categories	4 (3-4)	4 (4-4)	4 (4-4)	4 (4-4)
	I believe the recommendations are appropriate	4 (3-4)	4 (4-4)	4 (3.75-4.25)	4 (4-4)
	I used the suggestions to modify patient care	4 (4-4)	4 (4-5)	4 (4-5)	4 (4-5)
**Satisfaction, median (IQR)**					
	Overall, I am satisfied with the application	4 (4-4.75)	4 (4-5)	4 (4-5)	4 (3-4)
	It may be beneficial for patient care	4 (4-4.75)	4 (4-5)	4 (4-5)	4 (4-5)
	I will keep using it for patient care	4 (4-5)	4 (4-5)	4 (4-5)	4 (4-5)
	I would recommend this application	4 (3-4.75)	4 (4-5)	4 (4-5)	4 (4-5)

^a^Ranging from 1 to 5.

^b^Cronbach α for the Global questionnaire (24 items) is .93.

^c^NASF-AB: Núcleo Ampliado de Saúde da Família e Atenção Básica (multidisciplinary primary care support teams).

At the end-line assessment, health care professionals registered 4211 patients in the application’s database and 7960 consultations were performed by **primary health care** teams. Patients were predominantly female (2819/4211, 66.94%), median age was 55.0 (IQR 47.0-62.0). A total of 3993 (94.82%) patients were diagnosed with hypertension and 1028 (24.41%) were diagnosed with diabetes and there were 810 (19.24%) patients with diagnosis of both hypertension and diabetes.

#### Qualitative Analysis

In the focus groups, 13 physicians and 4 nurses participated (71% [12/17] female, median age 34 [IQR 31-39], ranging from 24 to 69 years). With regard to the difficulties dealing with the technology, the majority of participants reported some difficulties in the first days using the software, but no problems afterward. However, there was a statement of more difficulties among older professionals, who received help from the younger team members:

I started handling the software for patient assistance, as a nurse. I had no difficulties. Nowadays I help other professionals, such as doctors, who are more used to pen and paper. My unit has a doctor with 30 years experience, who had more difficulty adapting (using the software). As I showed him step by step and all functionalities of the software, he became interested and found it cool When he learnt that he could write patient prescriptions and print it using the software, he was very satisfied. So now, he prints the patient prescription and the exams, correctly. This way, he saves a lot of time.

Female, 37-year-old nurse, 13 years’ experience working as a primary care nurse

The structured clinical evaluation was perceived as an advantage, as exemplified below:

The questions we always ask (using the software). Are you feeling shortness of breath? Do you wake up out of breath? Sometimes in a rush, we forget (to ask those questions to the patient). But we always ask them (when using the software). It is as if you take the query and say: this is indispensable for you to ask to the patient. You always have to remember to ask about it. So, for me it's great. I will always remember to give counselling, for example, when the patient is a smoker, at the end there is always the ‘Did you talk about smoking?’ reminder. So there you will remember to tell the patient “Look, you have to stop smoking”. For me, this is very cool.

Female, 31-year-old physician, 4.5 years’ experience working as a primary care physician

There was no habit of measuring the blood pressure sitting, lying down and standing. After we started using the software, it became routine. We do not need to say anything. The nurse technician already measures the blood pressure of all patients that way.

Female, 31-year-old physician, 4.5 years’ experience working as a primary care physician

However, the time spent in the consultation was seen as a challenge for some participants. In some cases, they were forced by the health department to attend each patient for a maximum of 15 minutes, but this time was not sufficient when attending the patients using the software:

I take an average of 30 minutes per consultation, i.e. two consultations. This is the same I do with prenatal consultations for pregnant women.

Male, 39-year-old physician, 6 years’ experience working as a primary care physician

In other cases, there was an organizational issue in the primary care, which made it difficult to attend patients using the software:

My primary care center works as an emergency care. So we set aside a day to attend the patients from the project, which is a longer consultation. However, for example, we book ten patients from the program, but 20 show up; six from the project and the rest are patients with acute diseases. It is a challenge to attend the patients using the software, as there are other patients waiting. That makes it difficult.

Female, 32-year-old physician, 6 years’ experience working as a primary care physician

Often the CHWs were the ones who registered the patients in the software and their educational level was usually low in that region, so some health care professionals interviewed complained that spelling mistakes in patient names made them waste time trying to guess how the CHWs might have registered the patient’s name.

With regard to the clinical decision support functionality, all participants were consistent to affirm they were very satisfied with it. They considered the alerts and recommendations very useful for patient care, and reported that it influenced their patient care plan.

We usually don’t have time to calculate cardiovascular risk and kidney function. The software helps a lot.

Male, 39-year-old physician, 6 years’ experience working as a primary care physician

When attending a patient with high cardiovascular risk, who was taking 40 milligrams of simvastatin, and also amlodipine for hypertension, I received an alert to adjust the dosage of simvastatin to 20 milligrams, as amlodipine may increase the serum concentration of simvastatin and thus increase the risk of adverse effects. I had no idea, I learnt with the alert and changed patient prescription.

Female, 31-year-old physician, 4.5 years’ experience working as a primary care physician

There was a suggestion to improve the clinical decision support functionality, creating “red flags”:

I think the abnormal findings or the alerts could be in red. For example, the alert ‘This patient is in stage 3a renal failure’ should be in red.

Female, 39-year-old physician, 6 years’ experience working as a primary care physician

Concerning the ability to perform teleconsultations, the majority of participants said they have no time for it during the working hours. The ones who do it usually have to use their off-working hours:

I love to do teleconsultations, it helps the patients a lot. So much so that I am currently second (professional who requested the highest number of teleconsultations) in the state of Minas. We can help a lot people in primary care (using the teleconsultations). But I do it (the teleconsultation) at home; That's why it's hard work and a lot of people don't do it, there's no way to do it on the job.

Male, 69-year-old physician, 29 years’ experience working as a primary care physician

Finally, each software screen was projected and we discussed which variables they felt could be removed. All participants were consistent to affirm that all variables were very useful, except for patient admission, in which they had to fill in the date of admission and patient discharge, as well as the reason for admission. They considered these variables were not necessary, and their work could be reduced if they could be removed.

## Discussion

### Main Findings

This study described the development, usability, and satisfaction assessment of a web-based CDSS for diabetes, hypertension, and cardiovascular risk management in primary care. Its implementation in a low-resource setting, for users who were used to the paper-based patient records only, has shown to be feasible. Participants with a wide age range and experience in primary care found it to be usable, reported satisfaction, and attended almost 2000 patients using the system over 6 months and over 4000 patients by the end of follow-up.

With the adoption of the 2030 Agenda for Sustainable Development and the 17 Sustainable Development Goals [39], health systems and stakeholders worldwide are interested in innovative approaches to achieving universal health coverage objectives [15,40]. Although the Brazilian Constitution states that health care is free and provided by the public health system to all citizens, patients have unequal access to specialized health care services, especially in remote municipalities. Primary care physicians are often young people (less than 35 years of age) with little professional experience (less than 10 years of medical practice) [41]. Additionally, in those municipalities, there is a strong sense of professional isolation and high turnover of health professionals, which compromises the quality of health care [42]. In a recent meta-analysis, blood pressure control rates ranged from 43.7% to 67.5% in Brazilian patients [43]. Brazil is also one of the “most important examples of the alarming picture of diabetes in emergent societies,” with the fourth largest number of people with diabetes, and a poorer glycemic control rate than that observed in Europe and the United States. Even when a less stringent target was considered, only 48.5% of patients had an HbA1c level less than 8% [44]. In this context, digital health interventions may be very useful, especially those focusing on existing evidence-based health interventions, on the determinant layers of universal health coverage [15].

As CDSS recommendations evolve over time as evidence and the patient’s clinical state evolves, it provides a dynamic and personalized patient care plan that can be easily accessed by any health team member at any point in time [7], and may reduce the likelihood of unhelpful or risky prescriptions [45]. In this study, CDSS was implemented as part of a multifaceted strategy to enhance the delivery of evidence-based care in a resource-constrained area. It is innovative, as the majority of previous studies assessed the use of CDSS for hypertension and diabetes management in high-income countries [46]. Therefore, there is paucity of data on low- and middle-income countries and rural communities, which are the areas in most need and where the access to information technologies may be impaired. In our study, one-third of the total sample (34/96, 35%) and 52% of the doctors (13/25) did not use any form of technology for work before this research project. They did not use any kind of computerized physician order entry or electronic health records, so the implementation of the CDSS brought a significant change in workflow, which could be an important barrier for its usability [9,47]. Therefore, motivating these health care professionals and providing training in using the CDSS were challenging. Developing user-friendly interfaces and avoiding unnecessary information and excess of clicks were essential. Participants agreed that the interface was suitable. Making a tutorial video was important, but it did not replace the need for in-person training, and technical support was of utmost importance. Although the expert panel found the application intuitive, with no need for previous training, the health care professionals did not agree with that, which suggests different levels of technological skills.

With regard to the change in the workflow, health care professionals had to enter all the data items manually. It is reported that the burden of data entry may make them give up and abandon the CDSS [9]. At the same time, there is evidence that arduous data entry facilities adversely affect clinicians’ satisfaction [48], but the specificity level of computer-generated advice is known to highly influence the chance that physicians adhere to the advice [9]. To provide specific advice, is not possible to rely on limited data. A solution we found to try to overcome those barriers, facilitating the integration in the workflow and avoiding duplication of work, was to enable the health care professional to print the consultation using the CDSS and attach it to patient charts. The fact that it could be used by all primary care practitioners, each category with its allowances, and one complementing the other, with the inclusion of the educational groups and a patient management screen, was also important. Additionally, all the data on comorbidities and medications from a previous consultation did not need to be filled in again. Although the majority of primary care practitioners reported satisfaction with the application, doctors gave a low score for the variable “significant delays in daily routine” (median 2.0, IQR 2.0-3.0). Although the median score for doctors for “easy to be incorporated in work routine” was 4.0, 30% (7/23) of them choose a lower score (P25 [first quartile]=2.0). This was also reported in the focus groups. By contrast, when asked about reducing variables in the application, all professionals were consistent with the request to keep it the way it was.

Although the clinical impact assessment was not the focus at the early stages of implementation, there was improvement in outcomes related to hypertension and diabetes management, which are shown in another publication [12]. It is well established that managing the global cardiovascular risk is more important than only reducing blood pressure, glucose levels, or both [49], and this cardiovascular risk should drive preventive strategies. However, the majority of published CDSS did not include the assessment of cardiovascular risk. Choosing the cardiovascular risk calculator to include in the software was challenging, as no score was validated for the South American population [25]. We decided to use the Framingham score [32], as it was widely validated in different populations (European, Asian, Australian, and North American) and assessed important outcomes (coronary artery disease, stroke, peripheral artery disease, and heart failure) with no limitation in number of events (as in the case with the Atherosclerotic Cardiovascular Disease Risk Algorithm [ASCVD], which assessed only the first event) or restricted to fatal events only (as in the case with the Systematic Coronary Risk Evaluation [SCORE]) [50]. Additionally, it includes a number of variables that are easily obtained. In case total cholesterol and high-density lipoprotein cholesterol levels are not available yet, the risk can be estimated using another version of the score, in which BMI replaces the lipid levels [32,51].

### Limitations

This is study has some limitations. As was determined by the Ethics Committee, responses to the questionnaire were not identified, so we were not able to assess the results of feasibility, usability, and satisfaction according to the number of patients managed by each user. Additionally, there was no formal evaluation of the effectiveness. The software was part of a multifaceted intervention, whose results were published elsewhere [12], and it is not possible to access the impact of the software by itself. Meta-analyses suggest that CDSS with recommendations to both patients and health care professionals is more effective than providing CDSS to health care professionals only [8,52]. In this study, due to the high levels of illiteracy, we opted not to work directly with patients at that time.

### Success Factors

The majority of health care professionals (52/96, 54%) and 32% (8/25) of the doctors had no access to update an activity in the previous year. It is well established that CDSSs are designed to support decision making rather than making the decisions for the user, who makes the final decision based on his/her knowledge, experience, and scientific evidence provided by the CDSS or other sources [47]. Therefore, another important step was clinical training. Professionals had access to online classes, which were designed for each professional category and took into account their reality in terms of resources. Therefore, we believe that the opportunity of updating knowledge via online classes and by using the CDSS may have been one success factor, as has been already reported in the literature [7,8]. For those health care workers who had impaired access to continued education, there was a perception that the CDSS was a useful tool to improve patient care. It may have overcome the delays in daily routine and the perceived difficulty to be incorporated into the clinical workflow.

Management of diabetes and hypertension is a team effort, and many health care professionals may be involved [53,54], so we believe that the fact that all health care practitioners could access the CDSS, each on in their own role, was another important success factor, avoiding keeping patient care completely medical centered.

Careful monitoring of CDSS use rates, requesting feedback from users, and adjusting the application accordingly were other important success factors. Interventions lacking robust monitoring activities are unlikely to generate the impact expected from them and may lead to misjudgment of the digital health intervention as being not effective. As recommended by the WHO, robust monitoring and evaluation plans are essential to support effective implementation and potential intervention scale-up [15].

It is known that the level of adoption of a digital health intervention by users is “dependent on the end-users’ interaction with the technology and their belief/opinion that use of the technology will benefit their health” [16]. The responses to the questionnaire and the comments in the focus groups showed that this was probably another success factor in our study.

To ensure sustainability, the Brazilian Ministry of Health, MG state government, and municipal governments were involved from the beginning, as well as different stakeholders and local health professionals. The successful experience caught the Brazilian Ministry of Health’s interest, who in turn sponsored the continuation of the project’s activities for 2 years in the same region.

### Next Steps

We are planning to expand the project to primary care units in other towns, to include other diseases, and to implement in other settings. Our team is now working on the intervention refinement and software improvement, to subsequently conduct a cluster randomized controlled study to test the intervention implementation in other settings. The integration of the CDSS with the incipient electronic medical record of the Brazilian public health system (e-SUS) is a challenge, but we are currently working to overcome this barrier. Although there is limited transferability of the results to other settings, the lessons learnt may be useful for the implementation of other CDSSs in Brazil and around the world.

### Conclusion

In this study, a CDSS developed to assist the management of patients with hypertension or diabetes or both was applicable in the context of primary health care setting in a low income region, with good user’s satisfaction and potential to improve adherence to evidence-based practices.

## Appendix

Multimedia Appendix 1 Supplemental figure showing the dynamics of the system.

Multimedia Appendix 2 Supplemental figure showing the logic model of the study.

Multimedia Appendix 3 Supplemental table with detailed data of feasibility, usability and satisfaction assessment scores.

## Abbreviations

CDSS: clinical decision support system
CHW: community health worker
CKD-EPI: Chronic Kidney Disease Epidemiology Collaboration
FHS: family health service
HDI: Human Development Index
NASF-AB: Núcleo Ampliado de Saúde da Família e Atenção Básica
