# Supplementary figures and images for: Development and Implementation of a Decision Support System to Improve Control of Hypertension and Diabetes in a Resource-Constrained Area in Brazil: Mixed Methods Study
Source: J Med Internet Res. 2021 Jan 11;23(1):e18872. doi: 10.2196/18872 (PMC7834943; doi:10.2196/18872)

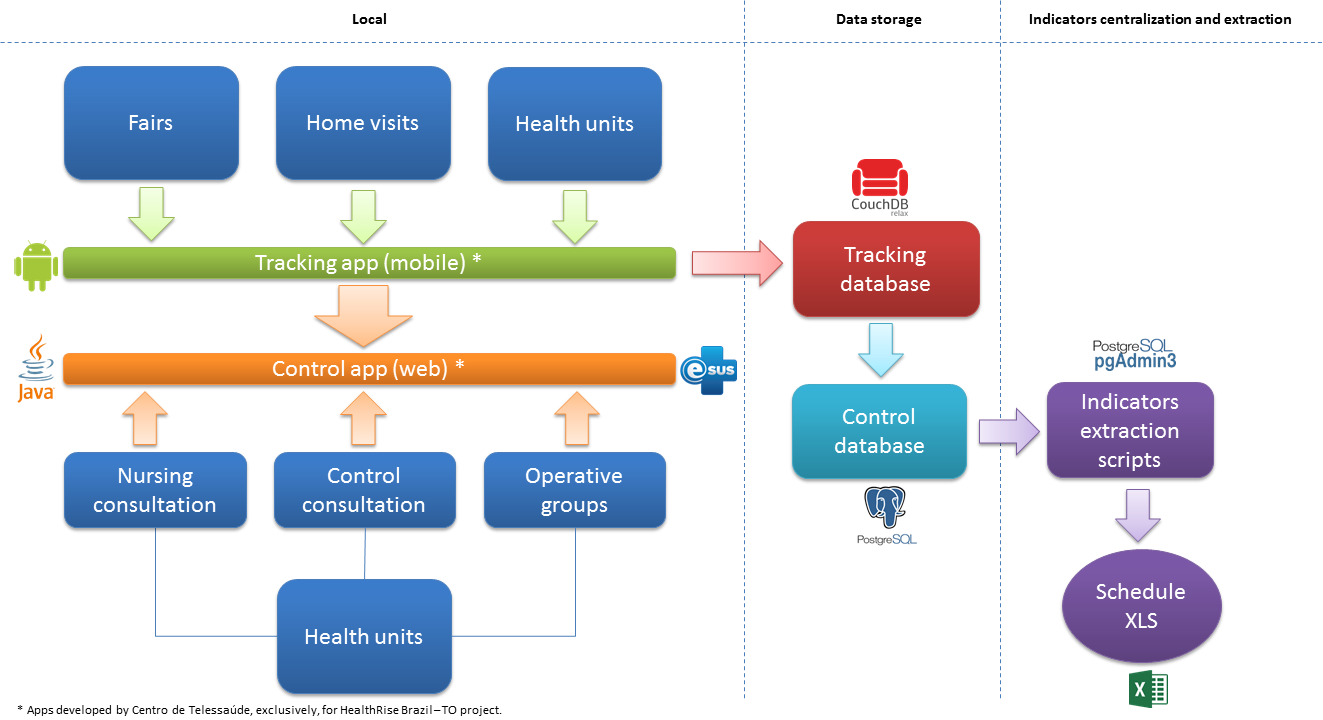

Supplement: Multimedia Appendix 1 [file jmir_v23i1e18872_app1.png]

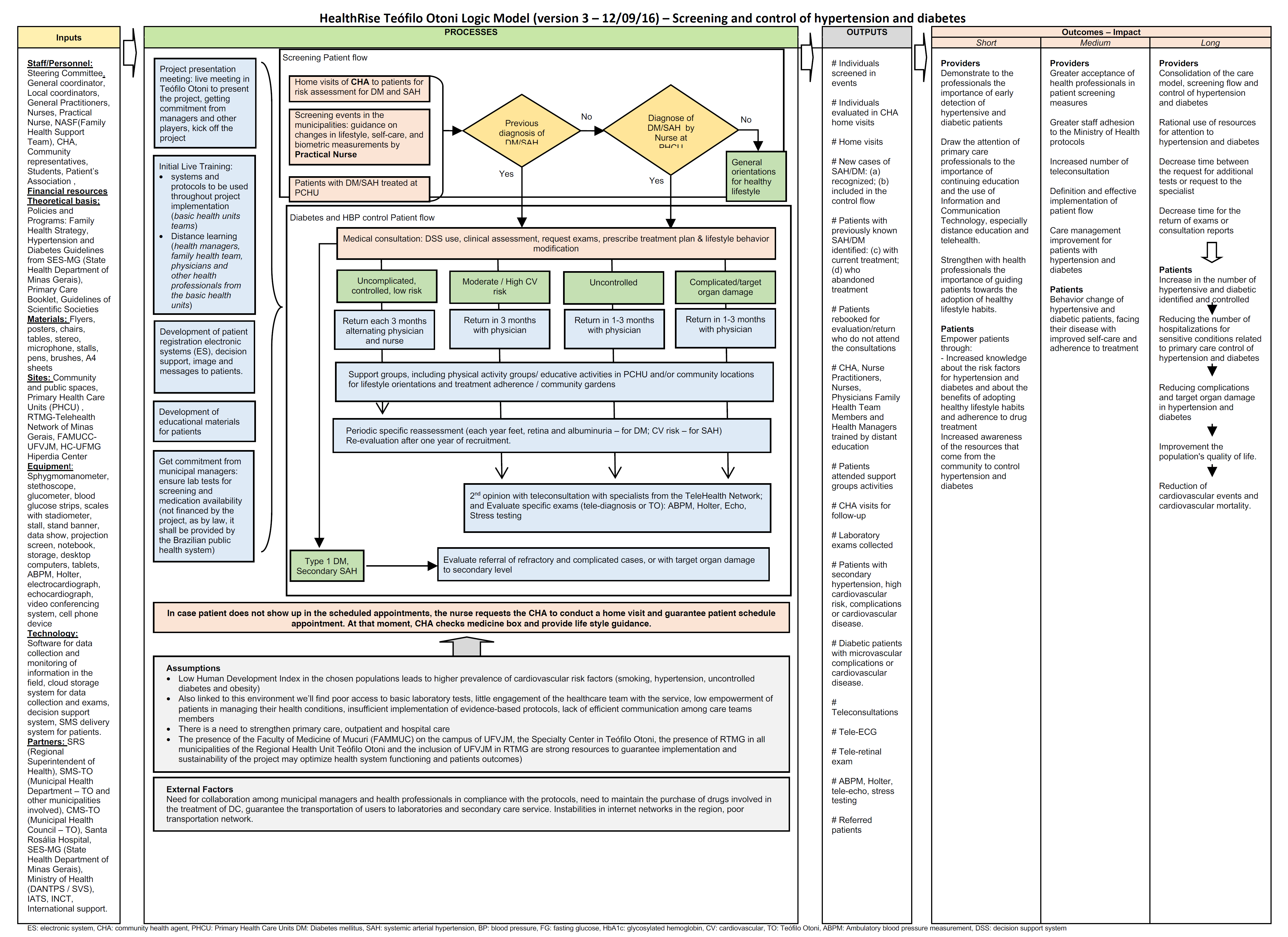

Supplement: Multimedia Appendix 2 [file jmir_v23i1e18872_app2.png]
