# Supplementary material for: Development and Implementation of a Decision Support System to Improve Control of Hypertension and Diabetes in a Resource-Constrained Area in Brazil: Mixed Methods Study
Source: J Med Internet Res. 2021 Jan 11;23(1):e18872. doi: 10.2196/18872 (PMC7834943; doi:10.2196/18872)
Supplement: Multimedia Appendix 3 [file jmir_v23i1e18872_app3.docx]

**Supplemental Table 1**. Feasibility, usability and satisfaction assessment scores^a^ (n=96).

| **Item** | **Physicians**  **(n=25)** | | **Nurses**  **(n=44)** | | **NASF**  **(n=27)** | | **Total** | |
| --- | --- | --- | --- | --- | --- | --- | --- | --- |
|  | **Mean (SD)** | **Median (IQR)** | **Mean (SD)** | **Median (IQR)** | **Mean (SD)** | **Median (IQR)** | **Mean (SD)** | **Median (IQR)** |
| **Feasibility** |  |  |  |  |  |  |  |  |
| The application can be used in the primary care setting, to assist patients with hypertension and/or DM | 4.21 (0.93) | 4 (4-5) | 4.48 (0.63) | 5.00 (4-5) | 4.42 (0.50) | 4.00 (4-5) | 4.39 (0.69) | 4.00 (4-5) |
| It is easy to be incorporated in work routine | 3.61 (1.23) | 4.00 (2-5) | 3.86 (0.91) | 4,00 (4-4) | 3.77 (0.86) | 4.00 (3-4) | 3.77 (0,98) | 4.00 (3-4) |
| The app does not cause significant delays in daily routine | 2.58 (1.28) | 2 (2-3) | 3.09 (1.15) | 3 (2-4) | 3.54 (0.86) | 4 (3-4) | 3.09 (1.16) | 3 (2-4) |
| **Usability** |  |  |  |  |  |  |  |  |
| My overall evaluation of the application is good | 4.00 (0.72) | 4 (3.25-4.75) | 4.23 (0.68) | 4 (4-5) | 4.12 (0.73) | 4 (4-5) | 4.14 (0.70) | 4 (4-5) |
| The application’s screens are easy to understand | 4.13 (0.54) | 4 (4-4) | 4.16 (0.71) | 4 (4-5) | 4.20 (0.50) | 4 (4-4.5) | 4.16 (0.61) | 4 (4-5) |
| I was able to find the information I was looking for | 4.00 (0.66) | 4 (4-4) | 3.95 (0.81) | 4 (4-4) | 4.08 (0.85) | 4 (4-5) | 3.84 (0.78) | 4 (4-4) |
| The definitions of comorbidities are clear and unambiguous | 4.04 (0.75) | 4 (4.4) | 4.14 (0.590 | 4 (4-4.75) | 3.96 (0.92) | 4 (4-5) | 4.06 (0.73) | 4 (4-4) |
| The application fields are easy to fill | 4.04 (0.64) | 4 (4-4) | 4.27 (0.59) | 4 (4-5) | 4.35 (0.63) | 4 (4-5) | 4.24 (0.62) | 4 (4-5) |
| The application has uniform and proper interface | 4.08 (0.50) | 4 (4-4) | 4.20 (0.55) | 4 (4-5) | 4.08 (0.94) | 4 (4-5) | 4.14 (0.67) | 4 (4-5) |
| The application is stable and no errors occur during the use | 3.09 (1.20) | 4 (2-4) | 3.09 (1.23) | 3 (2-4) | 3.00 (1.00) | 3 (2-4) | 3.07 (1.18) | 3 (2-4) |

Supplemental Table 1 Continued.

| **Item** | **Physicians**  **(n=25)** | | **Nurses**  **(n=44)** | | **“NASF”**  **(n=27)** | | **Total** | |
| --- | --- | --- | --- | --- | --- | --- | --- | --- |
|  | **Mean (SD)** | **Median (IQR)** | **Mean (SD)** | **Median (IQR)** | **Mean (SD)** | **Median (IQR)** | **Mean (SD)** | **Median (IQR)** |
| **Utility** |  |  |  |  |  |  |  |  |
| I believe that the application might improve patient care | 4.21 (0.59) | 4 (4-5) | 4.45 (0.66) | 5 (4-5) | 4.50 (0.58) | 5 (4-5) | 4.40 (0.63) | 4 (4-5) |
| Reading the recommendations of the application, I had access to new information on hypertension, DM and cardiovascular risk | 4.00 (0.72) | 4 (4-4) | 4.36 (0.61) | 4 (4-5) | 4.27 (0.72) | 4 (4-5) | 4.24 (0.68) | 4 (4-5) |
| The application was useful to calculate the cardiovascular risk of the patients | 4.13 (0.61) | 4 (4-4.75) | 4.32 (0.74) | 4 (4-5) | 4.19 (0.69) | 4 (4-5) | 4.23 (0.69) | 4 (4-5) |
| The application was useful to promote cardiovascular disease prevention actions among my patients | 4.25 (0.53) | 4 (4-5) | 4.34 (0.53) | 4 (4-5) | 4.12 (0.65) | 4 (4-5) | 4.26 (0.57) | 4 (4-5) |
| The application assisted me to treat my patients | 4.04 (0.69) | 4 (4-4) | 4.20 (0.70) | 4 (4-5) | 4.00 (0.57) | 4 (4-4) | 4.11 (0.66) | 4 (4-5) |
| The application assisted me to choose complementary exams for individual patients | 3.63 (0.88) | 4 (3-4) | 4.30 (0.64) | 4 (4-5) | 3.88 (0.86) | 4 (3.75-4.25) | 4.01 (0.81) | 4 (4-5) |
| The application was helpful to decrease referral to specialists | 3.54 (0.88) | 4 (2-4) | 3.66 (0.86) | 4 (3-4) | 3.85 (0.83) | 4 (4-4) | 3.69 (0.86) | 4 (3-4) |
| The application was useful to different professional categories among the primary care team | 3.71 (0.91) | 4 (3-4) | 3.89 (0.90) | 4 (4-4) | 4.00 (0.75) | 4 (4-4) | 3.87 (0.86) | 4 (4-4) |
| According to my previous knowledge, I believe the recommendations generated by the application are appropriate | 4.08 (0.65) | 4 (3-4) | 4.16 (0.53) | 4 (4-4) | 4.15 (0.46) | 4 (3.75-4.25) | 4.14 (0.54) | 4 (4-4) |
| I used the suggestions and reminders to modify patient care | 3.96 (0.86) | 4 (4-4) | 4.02 (0.80) | 4 (4-5) | 3.96 (0.77) | 4 (4-5) | 3.99 (0.80) | 4 (4-5) |

Supplemental Table 1 Continued.

| **Item** | **Physicians**  **(n=25)** | | **Nurses**  **(n=44)** | | **“NASF”**  **(n=27)** | | **Total** | |
| --- | --- | --- | --- | --- | --- | --- | --- | --- |
|  | **Mean (SD)** | **Median (IQR)** | **Mean (SD)** | **Median (IQR)** | **Mean (SD)** | **Median (IQR)** | **Mean (SD)** | **Median (IQR)** |
| **Satisfaction** |  |  |  |  |  |  |  |  |
| Overall, I am satisfied with the application | 3.96 (0.86) | 4 (4-4.75) | 4.18 (0.69) | 4 (4-5) | 4.08 (0.80) | 4 (4-5) | 4.10 (0.76) | 4 (3-4) |
| The application may be beneficial for patient care | 4.09 (0.79) | 4 (4-4.75) | 4.30 (0.59) | 4 (4-5) | 4.35 (0.56) | 4 (4-5) | 4.26 (0.64) | 4 (4-5) |
| If the application continues available for use after the end of the research project, I will keep using it for patient care | 3.71 (1.04) | 4 (4-5) | 4.30 (0.67) | 4 (4-5) | 4.15 (0.61) | 4 (4-5) | 4.11 (0.80) | 4 (4-5) |
| I would recommend using this application to my colleagues | 3.92 (0.88) | 4 (3-4.75) | 4.34 (0.65) | 4 (4-5) | 4.35 (0.56) | 4 (4-5) | 4.23 (0.71) | 4 (4-5) |
| **Cronbach's alfa - Global questionnaire (24 items)** | 0.93 | | | | | | | |

Values shown are median (interquartile range) except for Cronbach's alfa.

CV, cardiovascular; DM, diabetes mellitus; IQR, interquartile range; NASF, *Núcleo de Apoio à Saúde da Família* (multidisciplinary primary care support teams); SD, standard deviation.

^a^ Ranging from 1 to 5.
